# Stress Responses and Recovery in Student Athletes: Heart Rate Variability and Skin Conductance Patterns Across Cognitive Challenges—A Pilot Study

**DOI:** 10.3390/bs16060912

**Published:** 2026-06-03

**Authors:** Aylin Zekioğlu, Serdar Tok, Mert İşbilir, Said Enes Yılmaz, Erdal Binboğa, Nihal Dal

**Affiliations:** 1 Faculty of Sports Sciences, Manisa Celal Bayar University, Halil Erdoğan Cd., Ahmet Bedevi Mah., 45040 Manisa, Turkey; aylin.zekioglu@cbu.edu.tr (A.Z.); serdar.tok@cbu.edu.tr (S.T.); s.enes200@gmail.com (S.E.Y.); 2 Department of Physical Education and Sport Sciences, Democritus University of Thrace, University Campus, 69100 Komotini, Greece; mert_isbilir@hotmail.com; 3 Department of Biophysics, Faculty of Medicine, Ege University, 35100 Izmir, Turkey; erdal.binboga@ege.edu.tr

**Keywords:** mental stress, heart rate variability, skin conductance level, recovery

## Abstract

This pilot study aimed to examine autonomic nervous system responses in student-athletes using heart rate variability (HRV) and skin conductance level (SCL) during sequential cognitive stressors, including a modified Stroop test and a mental math task. Fifty-two university athletes (mean age = 21.7 ± 2.8 years) participated in this repeated-measures experimental study. HRV and SCL were recorded at baseline, during each stressor, and throughout the recovery periods following each task. Repeated-measures ANOVA revealed significantly elevated SCL across experimental conditions [F(2.09, 100.53) = 45.69, *p* < 0.001, η^2^ = 0.488], which remained above baseline during recovery, indicating sustained sympathetic activation. HR also increased significantly during stress exposure, indicating increased autonomic activation during cognitive stress [F(2, 103.11) = 14.30, *p* < 0.001, η^2^ = 0.230]. Contrary to the initial hypothesis, vagally mediated HRV indices, including RMSSD [F(2.73, 131.11) = 6.88, *p* < 0.001, η^2^ = 0.125] and HF power [F(2.85, 136.99) = 16.86, *p* < 0.001, η^2^ = 0.260], increased during cognitive stress, suggesting adaptive parasympathetic modulation rather than vagal withdrawal. These findings indicate that autonomic responses to cognitive stress in athletes may involve simultaneous sympathetic and parasympathetic activation rather than a simple vagal withdrawal response. Overall, the findings only partially supported the initial hypotheses. Despite limitations related to sample size and short recording periods, the present findings highlight the importance of evaluating HRV and SCL together when examining psychophysiological stress responses in athletic populations.

## 1. Introduction

In most real-life situations, such as work, athletics, or education, individuals encounter different stressors sequentially. Employees may face back-to-back deadlines, intense meetings, and complex problem-solving tasks, leaving little time for full recovery between stress episodes. Athletes also might have to face various physical and mental stressors that can give rise to serious autonomic nervous system (ANS) responses. These stressors may include rigorous training regimens, strategic planning, and intrapersonal and interpersonal challenges. Hence, it is necessary to examine individuals’ psychophysiological responses to successive stress episodes and their recovery processes following these episodes within well-controlled experiments, especially in athletic populations. Examining how sequential stress episodes affect ANS activity is essential, as stress can alter individuals’ ANS functioning and, eventually, physical and mental health. Monitoring autonomic responses to stress in athletes is important because repeated dysregulation of the autonomic nervous system may negatively influence recovery, performance, fatigue management, and psychological well-being. In athletic populations, impaired autonomic regulation has also been associated with maladaptive stress responses, overreaching, and reduced recovery capacity (Aubert et al., 2003; Kim et al., 2018; Järvelin-Pasanen et al., 2018).

Heart rate variability (HRV) is a non-invasive marker of cardiac autonomic regulation and reflects beat-to-beat variation in heart rhythm (European Task Force, 1996; Laborde et al., 2017). Time-domain indices such as SDNN and RMSSD, and frequency-domain indices such as LF and HF power, are commonly used to evaluate autonomic responses to physiological and psychological stressors (Kim et al., 2018; Thayer et al., 2012). However, HRV indices should be interpreted cautiously because they may be influenced by factors such as respiration, recording duration, and individual differences in fitness or stress regulation (Quintana & Heathers, 2014; Laborde et al., 2017). More recent methodological recommendations have further emphasized the importance of standardized HRV acquisition and interpretation in psychophysiological research (Laborde et al., 2017; Quintana & Heathers, 2014).

The HR of healthy individuals displays beat-to-beat variations resulting from fluctuations in ANS activity at the sinus node (Malliani et al., 1991; Thayer & Lane, 2009). HRV is evaluated using a variety of metrics, categorized into time-domain and frequency-domain measures. To assess HRV, the intervals between successive normal-to-normal (NN) beats, identified as R-R intervals on an ECG, are extracted from continuous ECG recordings. Subsequently, HRV parameters in both the time and frequency domains are calculated from these NN intervals.

In time-domain analysis, the most widely utilized parameters include the standard deviation of NN intervals (SDNN), the root mean square of successive NN interval differences (RMSSD), and the mean NN interval (NNMean). Frequency-domain analysis, which commonly employs fast Fourier transform (FFT), provides information regarding autonomic modulation patterns. Traditionally, LF power and the LF/HF ratio were interpreted as markers of sympathetic activity and sympathovagal balance (Pagani et al., 1986; Malliani et al., 1991). However, more recent evidence has questioned this interpretation, suggesting that LF power may not represent a pure index of sympathetic activity and should therefore be interpreted cautiously (Reyes del Paso et al., 2013; Laborde et al., 2017).

Previous research has demonstrated that HRV reflects the heart’s capacity to adapt to various stressors, whether physiological, psychological, or environmental (Kim et al., 2018; Thayer et al., 2012). Balıkçı et al. (2023) argue that depending solely on a single ANS marker may not provide a comprehensive understanding of the relationship between cognitive stress and the variability in autonomic activity. To better understand how cognitive stressors influence ANS activity, it is crucial to assess a variety of psychophysiological biomarkers that can accurately reflect the effects of stress on the autonomic system (Thayer et al., 2012; Kim et al., 2018; Quintana & Heathers, 2014).

In this respect, skin conductance level (SCL) is widely used as an electrodermal indicator of sympathetic nervous system activity (Benedek & Kaernbach, 2010; Critchley & Nagai, 2013). Because SCL reflects sweat gland activity, it provides complementary information to HRV when examining stress-related autonomic responses (Critchley, 2002). Therefore, using HRV and SCL together may offer a broader understanding of how student-athletes respond to sequential cognitive stressors (Balıkçı et al., 2023; Kim et al., 2018). Electrodermal activity (EDA), including SCL, is primarily regulated by cutaneous sympathetic nerve activity associated with eccrine sweat gland function (Critchley, 2002; Boucsein, 2012). Because EDA reflects changes in the electrical properties of the skin related to autonomic, emotional, and cognitive processing, it is considered a reliable psychophysiological biomarker of sympathetic activation during stress exposure (Critchley & Nagai, 2013). Therefore, SCL may provide valuable insight into cognitive and physiological stress responses associated with athletic training loads and mental demands.

Previous research demonstrated the utility of both HRV and SCL as an indicator of ANS’s responses to mental stressors. For example, a metanalysis by Castaldo et al. (2015) illustrated that acute mental stress might reduce HF power and increase the LF/HF ratio in healthy adults, which means that mental stress might give rise to parasympathetic withdrawal together with sympathetic activation. However, compared with HRV research, relatively fewer studies have specifically examined SCL responses to sequential cognitive stressors in athletic populations, indicating the need for further psychophysiological investigation using electrodermal measures.

Cognitive stressors may influence HRV through several physiological pathways, including attentional demand, emotional regulation processes, and respiratory alterations associated with mental effort. Acute cognitive stress is generally associated with increased cardiovascular activation and altered vagal modulation, although the direction and magnitude of HRV responses may vary depending on task characteristics and individual stress appraisal (Thayer et al., 2012; Kim et al., 2018). Although there is a substantial amount of literature on individuals’ psychophysiological responses to mental stressors, there has been comparatively less scientific focus on how different sequential mental stressors affect the ANS in athletes. Furthermore, the processes involved in psychophysiological recovery after experiencing successive stress episodes are not well understood. Previous research has primarily examined athletes’ physiological responses to physical stressors, leaving a gap in our understanding of how they respond to sequential mental stressors.

Recent studies have also emphasized the relevance of cognitive-performance tasks in athletic populations. For example, Teteris et al. (2025) used the Vienna Test System to examine training-induced changes in choice reaction time among U20 fencers, highlighting the importance of cognitive testing in sport-specific performance contexts. This supports the need to examine how athletes respond physiologically to sequential cognitive challenges.

In this study, we aimed to examine athletes’ psychological responses to sequential mental stressors, as measured by HR, HRV and SCL. Additionally, we sought to explore the psychophysiological recovery of athletes following these mental stressors. Drawing on existing research and theoretical frameworks, we hypothesized that mental stressors would elicit sympathetic activation and a withdrawal of parasympathetic activity, which we expected to manifest as a decrease in HRV and an increase in SCL. Furthermore, we anticipated that both HRV and SCL would return to baseline levels during the recovery periods that followed the cessation of the stressors. Therefore, examining simultaneous sympathetic and parasympathetic responses using multiple physiological markers may provide a more comprehensive understanding of stress adaptation in athletes.

## 2. Methods

### 2.1. Participants

52 undergraduate students (12 female), aged 19 to 24 (M = 21.7, SD = 2.8), participated in the study. Participants were recruited from university sports teams at Manisa Celal Bayar University through convenience sampling. Because the study focused specifically on student-athletes, the sample was not intended to be representative of elite or professional athlete populations. The sample included athletes from both team sports (e.g., soccer, basketball, volleyball) and individual sports (e.g., athletics, combat sports, and racket sports). All participants had regular training experience and were actively engaged in competitive sports during the data collection period. Participants were required to abstain from using any medications or ergogenic aids that can affect nervous and cardiovascular system functions and had no previously existing acute or chronic cardiovascular and psychiatric diseases. All experimental procedures were approved by the Manisa Celal Bayar University Faculty of Medicine Health Sciences Ethics Committee before data collection, and participants provided written informed consent prior to participation. A post hoc power analysis was conducted using G*Power 3.1 for repeated-measures ANOVA designs. Based on the observed medium effect size (η^2^ = 0.230), α = 0.05, and the present sample size (n = 52), the achieved statistical power exceeded 0.80, indicating adequate sensitivity for detecting within-subject effects.

### 2.2. Experimental Procedure

As HRV parameters can differ significantly from person to person, making comparisons between groups can be challenging. Hence, in accordance with the recommendations of Laborde et al. (2017), to overcome this issue, we adopted a within-subject repeated-measures design to analyze the HRV responses of the same participants during baseline, Stroop test, Stroop-recovery, mental math, and mental math-recovery. Baseline recordings and experimental procedures were also conducted in accordance with the methodological recommendations of Laborde et al. (2017) and Quintana and Heathers (2014), including short-term resting HRV assessment under standardized laboratory conditions, minimization of movement artifacts, and continuous acquisition of physiological signals throughout the experimental protocol. Each participant completed the experiment individually in a single session lasting approximately 25 min.

**Figure 1:**
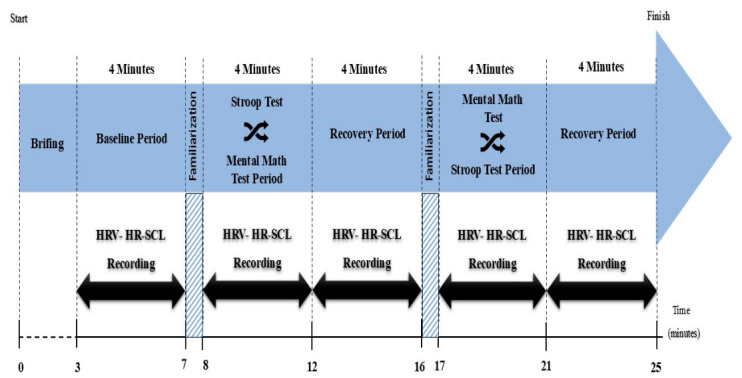
Schematic representation of the experimental procedure.

As illustrated in Figure 1, the experiment consisted of 5 periods, each lasting 4 min. Although the European Task Force (1996) traditionally recommends 5-min recordings for frequency-domain HRV analysis, previous psychophysiological studies and methodological recommendations have indicated that shorter recordings may still provide acceptable estimates for short-term autonomic responses under controlled experimental conditions (Quintana & Heathers, 2014; Laborde et al., 2017). Laborde et al. (2017) further suggested that approximately 4 min may represent a practical lower threshold for obtaining relatively stable LF estimates in short-term recordings under controlled experimental conditions, although such frequency-domain measures should still be interpreted cautiously. The 4-min segments used in the present study were selected to balance methodological rigor with the practical demands of repeated cognitive stress exposure and recovery assessment. In the first period of the experiment, we recorded participants’ baseline HR, HRV, and SCL responses. Before the experiment, the experimenters informed the participants about the procedure and task. Then, participants were allowed to practice five exercises for each experimental task to become familiar with the tasks. Afterward, participants completed the modified Stroop test or mental math tasks. The order of the tasks was counterbalanced using quasi-random assignment procedures to minimize potential order effects across participants. Participants rested for four minutes between the experimental tasks. Recovery in the present study refers to the short post-stressor period during which participants remained seated without engaging in any physical or cognitive activity. Therefore, the recovery periods were considered passive.

After the last experimental task was completed, we recorded participants’ post-stressor recovery responses, and the experiment ended. All measurements were conducted in a quiet laboratory environment at the Faculty of Sports Sciences. To minimize circadian influences on autonomic responses, experimental sessions were conducted during daytime hours under similar environmental conditions. Although no subjective perceived stress or mood scales were administered during the protocol, the selected cognitive tasks have been widely used in previous psychophysiological stress research to induce acute mental stress responses.

Figure 1 presents a schematic overview of the experimental procedure and recovery periods. The familiarization periods shown in Figure 1 are brief, task-specific practice sessions conducted before each cognitive task to ensure participants understood the instructions and task requirements.

### 2.3. Measures

#### HR and HRV Measurement

HR and HRV were continuously recorded throughout the experiment using a portable Nexus-10 Mark II system (Mind Media CV, Roermond, Herten, The Netherlands) in conjunction with its proprietary software (BioTrace V2018A, Mind Media BV, Herten, The Netherlands) and corresponding sensors. These sensors were wirelessly connected to the recording computer via Bluetooth. HR and HRV data were collected through electrocardiography (ECG) using a lead II configuration with three Ag/AgCl electrodes. One electrode was positioned below the right clavicle, another on the left side of the chest beneath the sixth rib, and the ground electrode was placed under the left clavicle. ECG signals were recorded at a 24-bit resolution with a sampling rate of 1024 Hz and processed using a 50 Hz notch filter.

### 2.4. Skin Conductance Level Measurement

For the analysis, the average SCL was determined via Nexus-10 Mark II (Mind Media CV, Roermond, Herten, The Netherlands) across the experimental sessions. SCL was measured using a skin conductance sensor (NX-GSR1A) at the index and ring finger of the non-dominant hand. All psychophysiological data were recorded with a 24-bit resolution at a frequency of 1024 Hz, except SCL (50 Hz notch filter). Data were sampled at a frequency of 32 Hz and subsequently stored on computer disks for further analysis. The recorded signals were expressed in microSiemens (μS).

#### 2.4.1. Stroop Test

The Stroop test was implemented to induce cognitive stress in student-athletes. In this task, participants were presented with color words (e.g., “RED,” “BLUE,” “GREEN”) displayed in incongruent ink colors (for instance, the word “RED” appearing in blue ink). They were instructed to identify the ink color while verbally disregarding the word. This challenge required both cognitive control and response inhibition, thereby increasing the participants’ mental load. The task was conducted over a duration of four minutes, during which participants’ HRV and SCL were continuously monitored to evaluate their autonomic responses to the stressor. 

Each participant was exposed to 48 word–color mismatch problems via SuperLab 4.0 software (Cedrus Corp, San Pedro, CA, USA). Word–color mismatch problems appeared on the screen for five seconds; at the end of the five seconds, the stimuli disappeared from the screen, and the subsequent stimuli was presented regardless of the participants’ response. A modified version of the Stroop task was used to increase cognitive interference and attentional demand under time pressure. The task was administered on a desktop computer using SuperLab 4.0 software.

#### 2.4.2. Mental Math Task

Participants completed a mental arithmetic task designed to induce cognitive load. The task involved performing a series of four-minute subtraction problems while their HRV and SCL responses were recorded under time constraints. The participants were asked to subtract 13 from a four-digit number. Each problem was presented on a screen for five seconds. After the participants’ correct answers, the experimenter manually presented the following problem.

#### 2.4.3. Psychophysiological Analysis

All psychophysiological data was analyzed via Nexus-10 Mark II recording (Mind Media CV; Roermond, Herten, The Netherlands) and its supplied software (Biotrace, Mind media B.V. Netherlands). The HRV of each participant was obtained based on the time series of peak-to-peak intervals that were immediately extracted from the ECG data. Before analyses, the peak-to-peak intervals were visually inspected for artifact rejection and unusual heartbeats (ectopic signals, premature signals), and then the corrected intervals were converted into the inter-beat interval (IBI) time series. Following the recommendations of the European Task Force (1996), the HRV time and frequency-domain analyses were performed by using the IBI time series. In the present study, we employed NNmean (ms), SDNN (ms), and RMSSD (ms) as the time-domain indices. The frequency-domain indices were computed via the FFT algorithm. In the present study, the power-spectrum density was measured for the primary frequency indices: the LF and HF power (ms^2^). The relationship between these bands (LF/HF ratio) was also analyzed. As for SCL analyses, mean SCL values were computed for each 4-min segment using the BioTrace^+^ software. The tonic component of SCL was derived following the methodology outlined by Boucsein (2012).

### 2.5. Statistical Analysis

We first log-transformed (Log 10) the HRV frequency-domain parameters to analyze the obtained data set to satisfy linear analysis requirements. Then, we conducted a series of repeated measures ANOVAs to explore whether HR, HRV, and SCL responses differed significantly among experimental conditions, namely the baseline, Stroop test, Stroop-recovery, mental math, and mental math-recovery. In reporting repeated measures ANOVA, we used a corrected degree of freedom via Greenhouse–Geisser estimates of sphericity if the assumption of sphericity was violated. We used the Bonferroni-corrected pairwise test after obtaining a significant main effect of repeated measures of ANOVA. We reported effect sizes using partial squared (η^2^) to determine the magnitude of observed differences. All statistical analyses were conducted using IBM SPSS Statistics (Version 26.0, IBM Corp., Armonk, NY, USA).

## 3. Results

The repeated measures ANOVA analysis revealed significant variations in SCL, HR, and HRV parameters across the five experimental conditions: Baseline, Stroop test, Stroop-recovery, mental math test, and mental math-recovery. The differences across the experimental conditions are presented below. Table 1 presents the results of the repeated measures ANOVA.

**Figure 2:**
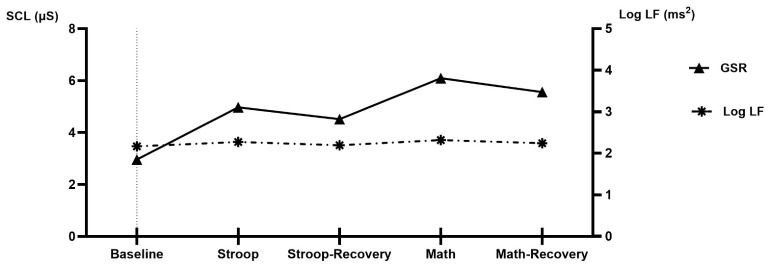
Changes in SCL and log LF across experimental periods. Abbreviations used in the figure: Base = Baseline; Stroop Rec = Stroop-Recovery; Math = Mental Math; Math Rec = Mental Math-Recovery.

As shown in Figure 2, The SCL values showed a significant main effect across conditions [F (2.09, 100.53) = 45.69, *p* < 0.001, η^2^ = 0.488]. As demonstrated in Table 1 specifically, SCL was significantly higher during the Stroop and mental math test conditions compared to baseline (*p* < 0.001), indicating increased sympathetic nervous system activation during these cognitive tasks. Additionally, significant differences were observed between the Stroop-recovery and mental math test conditions (*p* < 0.001), suggesting an incomplete return to baseline levels before the second stressor was introduced. The relatively large standard deviation values observed for SCL may reflect substantial interindividual variability in electrodermal responses to cognitive stressors, which is commonly reported in psychophysiological research (Benedek & Kaernbach, 2010; Critchley & Nagai, 2013).

**Figure 3:**
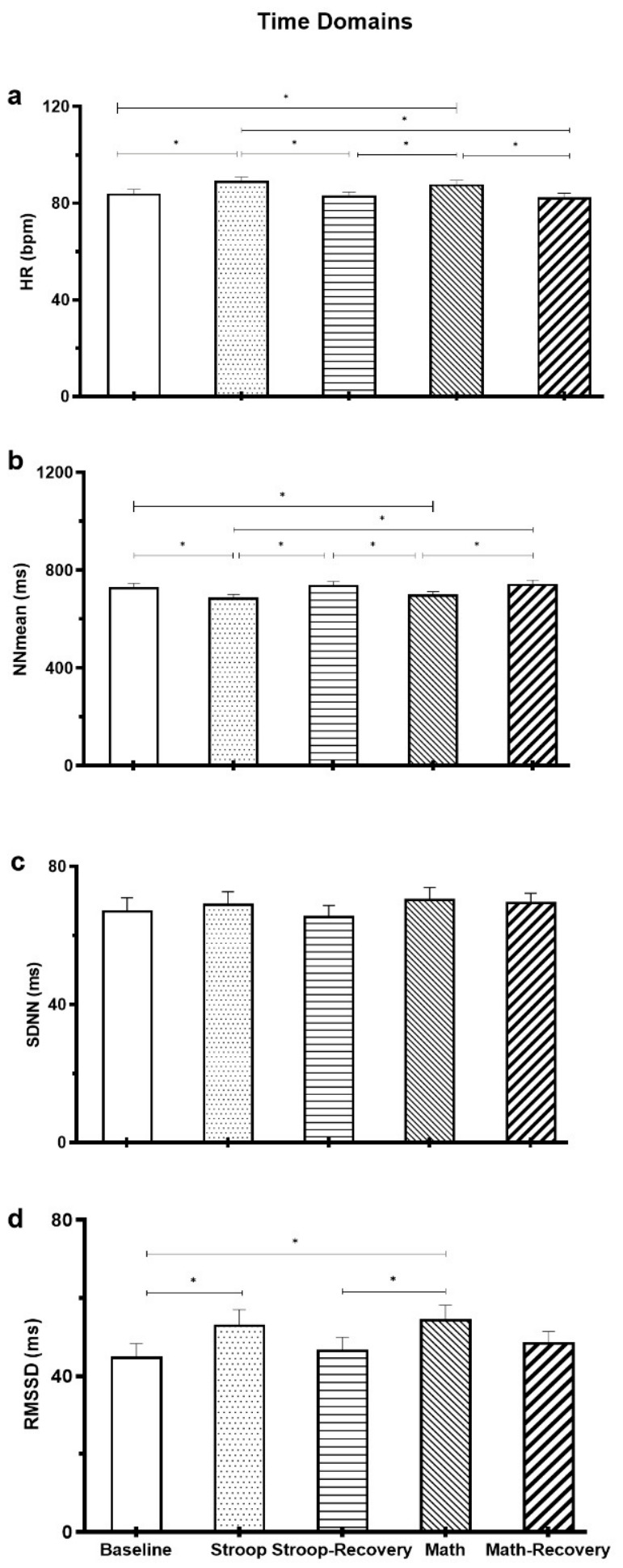
Changes in HR and HRV time-domain parameters (NNmean, SDNN, RMSSD) across experimental periods. (**a**) HR; (**b**) NNmean; (**c**) SDNN; (**d**) RMSSD. Abbreviations used in the figure: Base = Baseline; Stroop Rec. = Stroop-Recovery; Math. = Mental Math; Math Rec. = Mental Math-Recovery; * *p* < 0.05.

Similarly, HR exhibited significant changes across conditions [F (2.14, 103.11) = 14.30, *p* < 0.001, η^2^ = 0.230]. HR increased significantly from the baseline to the Stroop test (*p* < 0.001) and from the baseline to the mental math test (*p* < 0.001). The recovery phases, particularly the mental math recovery, showed a decrease in HR, indicating a partial return to baseline. Figure 3a shows HR differences during the entire experiment.

As demonstrated in Table 1 and Figure 3b–d, HRV parameters also demonstrated significant variations. As illustrated in Figure 3b, the time domain of NNmean, which represents the average interbeat interval, was significantly different across conditions [F (2,25, 108.23) = 18.64, *p* < 0.001, η^2^ = 0.280]. The Stroop test significantly reduced NNmean compared to baseline (*p* < 0.001), reflecting increased sympathetic dominance during cognitive stress. Moreover, NNmean was significantly lower during the Stroop test than during the mental math test (*p* < 0.001), suggesting that different cognitive tasks may induce varying levels of autonomic activation.

Regarding time-domain HRV measures, RMSSD was significantly influenced by conditions [F (2.73, 131.11) = 6.88, *p* < 0.001, η^2^ = 0.125]. As shown in Figure 3d, RMSSD significantly increased from baseline to the Stroop test (*p* = 0.011), indicating a short-term rise in parasympathetic modulation. Additionally, a significant increase was observed between the mental math test and the mental math-recovery phase (*p* = 0.002), suggesting that recovery after cognitive stressors may lead to enhanced vagal tone. However, as Figure 3c illustrates, SDNN did not show a statistically significant effect across the five conditions [F (4, 192) = 0.754, *p* = 0.548, η^2^ = 0.015]. This result indicates that the overall variability in HR, as measured by SDNN, remained relatively stable despite the application of cognitive stressors and recovery periods. Table S1 also demonstrates the results of the multiple comparisons with Bonferroni corrections as post hoc.

Detailed Bonferroni post hoc comparisons are provided in the Supplementary Materials (Table S1).

**Figure 4:**
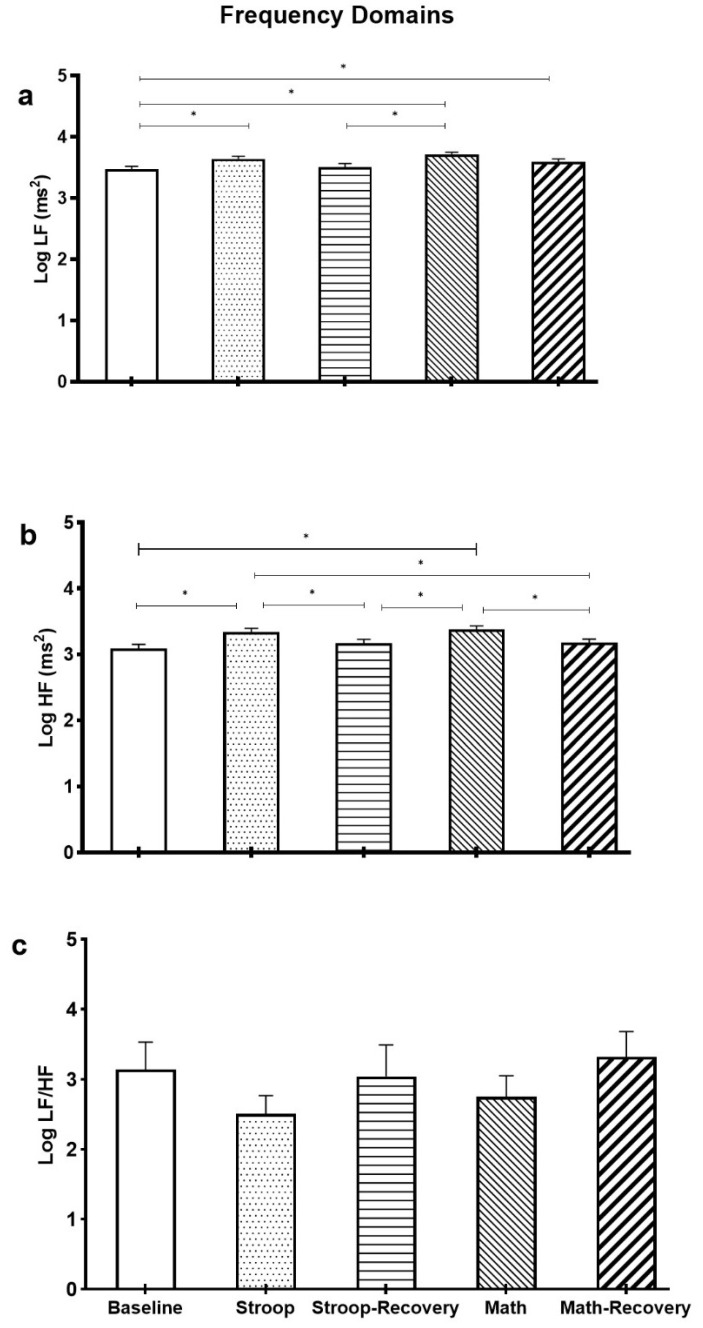
Changes in HRV frequency-domain parameters (log LF, log HF, and LF/HF ratio) across experimental periods. (**a**) HR; (**b**) NNmean; (**c**) SDNN; Abbreviations used in the figure: Base = Baseline; Stroop Rec. = Stroop-Recovery; Math = Mental Math; Math Rec. = Mental Math-Recovery. Log LF = logarithmically transformed low-frequency HRV power; Log HF = logarithmically transformed high-frequency HRV power. * *p* < 0.05.

Frequency-domain HRV measures further supported these findings. Log LF power exhibited significant differences across conditions [F (3.28, 154.36) = 8.81, *p* < 0.001, η^2^ = 0.158]. Specifically, as illustrated in Figure 4a, Log LF power was significantly higher in the mental math test compared to baseline (*p* = 0.007) and the Stroop-recovery period (*p* < 0.001), indicating increased sympathetic modulation during the cognitive task. Similarly, as demonstrated in Figure 4b, Log HF power was significantly influenced by condition [F (2.85, 136.99) = 16.86, *p* < 0.001, η^2^ = 0.260], with higher values observed in the Stroop and mental math tests compared to baseline (*p* < 0.001), reflecting elevated parasympathetic activity during stress.

The LF/HF ratio, an indicator of autonomic balance, did not show a statistically significant main effect across conditions [F (2.98, 143.41) = 1.77, *p* = 0.155, η^2^ = 0.036], suggesting that while individual frequency components (LF and HF) changed significantly in the present study, their relative balance remained stable. Figure 4c shows the LF/HF changes during the entire experiment.

## 4. Discussion

This study explored the effects of sequential mental stressors on HRV and SCL in student-athletes. Our findings reveal that engaging with a modified Stroop word–color test and performing a mental math task elicited significant responses from the ANS. As anticipated, SCL increased throughout the stress episodes and did not return to baseline levels even during the recovery phases. However, contrary to our expectations, vagally mediated HRV—reflected by elevated RMSSD and HF power—rose during the stress periods but decreased during recovery periods. Overall, the results indicate that both HRV and SCL respond to acute mental stressors during stress and the subsequent recovery periods. The simultaneous increase in sympathetic-related electrodermal activation and vagally mediated HRV indices may also be interpreted within the framework of the “autonomic space” model proposed by Berntson et al. (1991), which suggests that sympathetic and parasympathetic branches of the autonomic nervous system may coactivate rather than operate in a strictly reciprocal manner.

The first finding we want to address is the elevated vagal activity observed during stress periods. The observed HRV response, characterized by increased parasympathetic activity during stress, is inconsistent with findings from some previous studies (Kim et al., 2018; Usui & Nishida, 2017). However, these findings indicate elevated RMSSD and HF power in response to sequential acute mental stress, suggesting adaptive autonomic modulation, although alternative explanations should be considered.

HRV measures, particularly HF power and RMSSD, are strongly influenced by respiration patterns and breathing frequency (Laborde et al., 2017; Quintana & Heathers, 2014). Since respiration was not controlled in the present study, the observed increases in vagally mediated HRV indices should be interpreted cautiously, as they may partially reflect breathing-related influences rather than purely autonomic changes.

Elevated vagal activity during stress exposure might be explained through several theoretical frameworks. First, the compensatory (homeostatic) mechanism suggests that the ANS dynamically regulates physiological responses to maintain homeostasis, wherein vagal activation may increase as a counter-regulatory response to heightened sympathetic arousal (Berntson et al., 1991). Second, the biphasic stress response and arousal theory propose that moderate levels of stress can enhance physiological adaptability, wherein an initial sympathetic activation may be followed by increased parasympathetic activity as part of an adaptive recovery process (McEwen, 1998; Yerkes & Dodson, 1908). Third, from the perspective of psychological resilience and cognitive appraisal, the interpretation of stressors as a challenge rather than a threat might modulate autonomic responses. According to the biopsychosocial model of challenge and threat proposed by Blascovich et al. (2003), individuals who perceive a task as achievable exhibit an adaptive cardiovascular response, characterized by sustained vagal activity. This physiological response promotes efficient regulation, supporting optimal functioning during the task (Blascovich & Mendes, 2010; Blascovich & Tomaka, 1996). These findings may also be interpreted within the framework of cognitive appraisal theories, suggesting that athletes may perceive stressors as challenges rather than threats, leading to more adaptive autonomic responses.

Further, the model also asserts that challenge and threat appraisal might be associated with cardiovascular arousal indices such as HR, pre-ejection period, cardiac output, and total peripheral resistance. Therefore, university-level student-athletes may become more familiar with repeated cognitive and competitive stress exposure, which could influence autonomic regulation patterns during experimentally induced stress.

The observed results in the present study might stem from the physical and psychological characteristics of university-level student-athletes rather than elite athletic populations. University-level student-athletes may exhibit relatively higher physical fitness and stress adaptation capacities compared with non-athletic populations, although these characteristics may differ substantially from elite athletes (Aubert et al., 2003). This connection may help clarify the findings presented in this study. Previous research indicates that HRV is decreased in response to a modified version of the Stroop test within an older, nonathletic population characterized by lower physical activity levels (Delaney & Brodie, 2000). This evidence may lend support to our findings, which reveal a vagal rebound during exposure to a similar test. Moreover, the HR values observed throughout the experimental protocol (approximately 82–89 bpm) may indicate relatively low-intensity physiological activation in trained athletes, corresponding to Zone 1 autonomic responses. This finding may suggest that the cognitive stressors used in the present study elicited moderate cognitive demand without producing excessive cardiovascular strain. Similar findings have been reported in recent sport-performance research examining cognitive and attentional demands in athletic populations (Teteris et al., 2025).

Although emotional regulation and cognitive appraisal processes may theoretically influence autonomic stress responses, these variables were not directly measured in the present study. Therefore, any interpretation regarding emotional regulation capacity should be considered speculative.

The PNS, primarily regulated by the vagus nerve, acts as a “brake” to counteract the heightened sympathetic arousal associated with the stress response (Browning, 2019; Thayer & Lane, 2009). This activation of the parasympathetic system helps moderate and buffer the physiological effects of stress, potentially serving as a protective mechanism against excessive or prolonged arousal, which can become maladaptive if not managed properly (Browning, 2019). Additionally, the observed decrease in vagal activity during the recovery period supports this understanding, as the vagal “brake” is released when the body returns to a state of homeostasis.

The sustained increase in SCL observed in the current study corroborates previous research demonstrating that acute psychological stress elicits robust sympathetic arousal, as measured by electrodermal activity (Järvelin-Pasanen et al., 2018; Kim et al., 2018). The lack of recovery in SCL during the post-stress period suggests that the student-athletes experienced perseverative cognitive processing and prolonged physiological activation even after the cessation of the stressors. An elevated level of SCL throughout the experiment might stem from several factors, such as ANS dynamics and certain psychological qualities (Critchley, 2002; Critchley & Nagai, 2013). Accordingly, SCL is controlled by the sympathetic branch of the ANS and, contrary to HRV, does not reflect the parasympathetic branch of the ANS. Hence, once SCL increases as a result of the eccrine sweat glands’ response to stress, as there is no parasympathetic counter-regulation that can suppress sweat gland activity, it might require a more prolonged duration to regress to its baseline level.

Additionally, although our study design did not include specific individual differences (e.g., personality traits, coping strategies, emotional intelligence) as potential moderators, those factors may have influenced the observed patterns of sustained SCL response.

## 5. Conclusions

The results of the present study suggested that sequential cognitive stressors significantly influenced autonomic nervous system responses in student-athletes. Consistent with the initial hypotheses, SCL increased during cognitive stress exposure and remained elevated during recovery periods, indicating sustained sympathetic activation. However, contrary to expectations, vagally mediated HRV indices such as RMSSD and HF power also increased during stress exposure, suggesting adaptive parasympathetic modulation rather than vagal withdrawal. These findings indicate that the sympathetic and parasympathetic branches of the autonomic nervous system may coactivate during cognitive stress in university-level student-athletes. Therefore, the findings of the present study only partially supported the initial hypotheses.

The present findings suggest that sequential cognitive stressors may produce simultaneous increases in electrodermal sympathetic activation and vagally mediated HRV indices in university-level student-athletes. The observed autonomic response pattern indicates that cognitive stress responses may involve coactivation of sympathetic and parasympathetic processes rather than a simple reciprocal shift between activation and recovery states.

The present study has several limitations. First, the small sample size may restrict the generalizability of the findings. The unequal sex distribution of the sample may represent a limitation. Previous literature has demonstrated that autonomic regulation and HRV responses may differ according to sex, particularly in young adults, which may influence stress-related vagal responses (Thayer et al., 2012; Koenig & Thayer, 2016). Because the present study was not designed or powered to examine sex-specific effects, these findings should be interpreted cautiously. Second, respiration was not controlled during the experiment, a known factor that influences HRV indices, particularly RMSSD and HF power. Additionally, pre-experimental psychological stress levels, sleep quality, and daily life stressors were not objectively controlled prior to laboratory sessions, which may have influenced HRV and SCL responses. Third, the relatively short recording periods (4 min) may limit the stability of frequency-domain HRV measures. Fourth, the use of laboratory-based cognitive stressors may not fully reflect real-world sport-specific stress conditions. Furthermore, the absence of a sedentary or non-athlete comparison group limits the ability to determine whether the observed autonomic response patterns were specifically associated with athletic status. Additionally, the present sample consisted specifically of university-level student-athletes recruited through convenience sampling. Therefore, the findings should not be generalized to elite, professional, or broader athletic populations without further evidence from more diverse and representative samples. Additionally, the current research did not include variables that could influence the relationship between autonomic stress responses and mental stress, such as personality traits, coping strategies, and emotional intelligence. Furthermore, although emotional regulation and cognitive appraisal processes were discussed as possible explanatory mechanisms, these psychological variables were not directly measured in the present study.

## Figures and Tables

**Table 1:** Results of the Repeated Measures ANOVA for Time- and Frequency-Domain Parameters.

Repeated Measure ANOVA													
Variables	Baseline		Stroop		Stroop-Recovery		Math		Math-Recovery		F	*p*	η^2^
	Mean	SD	Mean	SD	Mean	SD	Mean	SD	Mean	SD			
SCL	2.96	4.71	4.97	6.95	4.52	5.73	6.09	6.50	5.56	5.73	45.69	<0.001 *	0.488
HR (bpm)	84.02	11.65	89.23	11.24	83.07	10.79	87.89	11.00	82.53	11.49	14.30	<0.001 *	0.230
NNmean (ms)	731.43	101.63	687.68	93.13	739.64	98.48	699.84	90.00	744.86	99.74	18.64	<0.001 *	0.280
SDNN (ms)	67.25	25.77	69.25	23.77	65.70	21.02	70.67	22.37	69.70	17.71	0.754	0.548	0.015
RMSSD (ms)	45.00	23.38	53.25	26.49	46.83	21.63	54.70	24.21	48.73	18.74	6.88	<0.001 *	0.125
Log LF (ms^2^)	3.47	0.34	3.64	0.30	3.51	0.38	3.71	0.28	3.59	0.34	8.81	<0.001 *	0.158
Log HF (ms^2^)	3.09	0.42	3.34	0.38	3.17	0.40	3.38	0.37	3.18	0.35	16.86	<0.001 *	0.260
LF/HF	3.14	2.75	2.51	1.79	3.04	3.17	2.75	2.09	3.32	2.55	1.77	0.155	0.036

Abbreviations: SCL = skin conductance level; HR = heart rate; NNmean = mean normal-to-normal (NN) HR intervals; SDNN = standard deviation of NN intervals; RMSSD = root mean square of successive NN interval differences; HF = high-frequency power; LF = low-frequency power; LF/HF = ratio of low-frequency power to high-frequency power; SD = standard deviation, *: *p* < 0.05.

## Data Availability

The data that support the findings of this study are available from the corresponding author upon reasonable request.

## Supplementary Materials

The following supporting information can be downloaded at: https://www.mdpi.com/article/10.3390/bs16060912/s1, Table S1. Results of the Bonferroni Test as Post Hoc.
