# Supplementary material for: Stress Responses and Recovery in Student Athletes: Heart Rate Variability and Skin Conductance Patterns Across Cognitive Challenges—A Pilot Study
Source: Behav Sci (Basel). 2026 Jun 3;16(6):912. doi: 10.3390/bs16060912 (PMC13295499; doi:10.3390/bs16060912)
Supplement: Supplementary file 1 [file behavsci-16-00912-s001.zip › behavsci-4277196-supplementary.pdf]

Supplementary Material

Table S1. Results of the Bonferroni post-hoc comparisons across experimental conditions.

Multiple Comparison								
**Variables**	**A**		**B**		**C**		**D**	
	**t**	**p**	**t**	**p**	**t**	**p**	**t**	**p**
**SCL**	-2.00	**< .001***	-1.55	**< .001***	-3.12	**< .001***	-2.59	**< .001***
**HR (bpm)**	-5.20	**.010***	0.951	1.00	-3.86	**.019***	1.49	0.88
**NNmean (ms)**	43.74	**< .001***	-8.21	1.00	31.58	**.019***	-13.43	0.50
**RMSSD (ms)**	-8.25	**.011***	-1.83	1.00	-9.70	**.002***	-3.73	0.60
**Log LF (ms^2)**	-0.164	**.007***	-0.033	1.00	-0.238	**< .001**	-0.121	0**.02***
**Log HF (ms^2)**	-0.0249	**< .001***	-0.083	0.08	-0.287	**< .001**	-0.0089	0.22

Multiple Comparison						
**Variables**	**E**		**F**		**G**	
	**t**	**p**	**t**	**p**	**t**	**p**
**SCL**	0.44	**.36**	-1.11	**< .001***	-0.58	0.54
**HR (bpm)**	6.15	**< .001***	1.33	1.00	6.66	**< .001***
**NNmean (ms)**	-51.96	**< .001***	-12.15	0.23	-57.17	**< .001***
**RMSSD (ms)**	6.41	0.14	-1.45	1.00	4.52	1.00
**Log LF (ms^2)**	0.131	0.17	-0.074	0.40	0.043	1.00
**Log HF (ms^2)**	0.166	**.002***	-0.038	1.00	0.0160	**0.014***

Multiple Comparison						
**Variables**	**H**		**I**		**J**	
	**t**	**p**	**t**	**p**	**t**	**p**
**SCL**	-1.56	**< .001***	-1.03	**< .001***	0.53	**0.02***
**HR (bpm)**	-4.81	**< .001***	0.54	1.00	5.36	**< .001***
**NNmean (ms)**	39.80	**< .001***	-5.21	1.00	-45.01	**< .001***
**RMSSD (ms)**	-7.86	**.02***	-1.89	1.00	5.97	0.16
**Log LF (ms^2)**	-0.205	**.002***	-0.088	0.74	.00117	0.25
**Log HF (ms^2)**	-0.204	**< .001***	-0.006	1.00	0.198	**.002***
